# Multiomic analysis of stretched osteocytes reveals processes and signalling linked to bone regeneration and cancer

**DOI:** 10.1038/s41536-021-00141-3

**Published:** 2021-06-07

**Authors:** Lívia Santos, Aslihan Ugun-Klusek, Clare Coveney, David J. Boocock

**Affiliations:** 1 Department of Sport Science, Sport, Health and Performance Enhancement Research Centre (SHAPE), School of Science and Technology, Nottingham Trent University, Nottingham, UK; 2 Department of Biosciences, Centre for Health, Ageing and Understanding Disease (CHAUD), School of Science and Technology, Nottingham Trent University, Nottingham, UK; 3 John van Geest Cancer Research Centre, Centre for Health, Ageing and Understanding Disease (CHAUD), School of Science and Technology, Nottingham Trent University, Nottingham, UK

**Keywords:** Fracture repair, Bone cancer, Regenerative medicine

## Abstract

Exercise is a non-pharmacological intervention that can enhance bone regeneration and improve the management of bone conditions like osteoporosis or metastatic bone cancer. Therefore, it is gaining increasing importance in an emerging area of regenerative medicine—regenerative rehabilitation (RR). Osteocytes are mechanosensitive and secretory bone cells that orchestrate bone anabolism and hence postulated to be an attractive target of regenerative exercise interventions. However, the human osteocyte signalling pathways and processes evoked upon exercise remain to be fully identified. Making use of a computer-controlled bioreactor that mimics exercise and the latest omics approaches, RNA sequencing (RNA-seq) and tandem liquid chromatography-mass spectrometry (LC-MS), we mapped the transcriptome and secretome of mechanically stretched human osteocytic cells. We discovered that a single bout of cyclic stretch activated network processes and signalling pathways likely to modulate bone regeneration and cancer. Furthermore, a comparison between the transcriptome and secretome of stretched human and mouse osteocytic cells revealed dissimilar results, despite both species sharing evolutionarily conserved signalling pathways. These findings suggest that osteocytes can be targeted by exercise-driven RR protocols aiming to modulate bone regeneration or metastatic bone cancer.

Regenerative rehabilitation (RR) is a growing area of regenerative medicine that offers the possibility of using exercise to improve clinical outcomes^1^. Patients with bone fractures but depicting normal protective sensation can undertake a weight-bearing exercise to augment their endogenous tissue regeneration capacity^2^. Individuals with chronic diseases like osteoporosis or cancer can benefit from exercise as well since this limit the amount of bone loss and improve survival rates, respectively^3,4^.

Exercise supports bone regeneration and mechanically stimulated osteocytes play a critical role in it by mediating bone anabolism^5^ and preserving cell viability^6^. With regards to bone anabolism, mechanically stimulated osteocytes secrete fewer of the paracrine factors RANKL and SOST allowing osteoblasts to mature and secrete extracellular matrix (ECM) leading to the formation of new bone^7–9^. This is evident in a mechanical loading model comprising osteocytic cells in 3D in direct coculture with osteoblasts in 2D that showed higher expression of type I collagen at day 1 and 5 of stimulation^9^. As for viability, mechanical stimulation prevents osteocyte apoptosis and downstream events like enhanced bone resorption and loss of bone mass^10,11^.

Besides supporting bone regeneration, mechanically stimulated osteocytes can limit the progression of metastatic breast cancer to bones^12,13^. This is demonstrated by in vivo studies where cyclic compression of mice tibia inhibited the growth and osteolytic capacity of breast tumour cells^12^ and in vitro where mouse osteocytic cells mechanically stimulated by fluid flow shear stress (FFSS) reduced breast cancer extravasation^13^.

The signalling pathways evoked by mechanically stimulated osteocytes implicated in bone regeneration are PI3K/AKT^14^, Wnt/β-catenin^15^ and TNF^16^. For example, cyclic stretch upregulates osteocyte PI3K/AKT leading to early activation of Wnt/β-catenin signalling^14^, which triggers the release of proteins like Wnt1^15^ prompting osteoblasts to produce new bone^17^, whereas shear stress inhibits the circulation of the apoptotic molecule, TNF-α^16^, which helps to preserve osteocyte viability and bone mass. Despite these findings, a comprehensive understanding of the molecular signalling evoked by mechanically stimulated osteocyte in both bone regeneration and bone cancer is missing.

A study on mechanotransduction and genome integrity demonstrated that adequate matrix elasticity protects the genome integrity via DNA repair pathways^18^ which is paramount to limit both cancer initiation and progression. We hypothesise that mechanically stimulated osteocytes activate similar pathways enabling them to modulate cancer progression. Therefore, this study aims to ascertain whether cyclic tension activates signalling pathways linked to genome integrity and identify any others capable of modulating both bone regeneration and cancer. For that, human osteocyte-like cells will be cultured under cyclic tension and the transcriptome and secretome mapped by RNA-seq and LC-MSMS, respectively. With this, we expect to expand current knowledge about the role of exercise or mechanically stimulated osteocytes in bone regeneration and cancer and contribute to the development of more informed RR protocols aiming to manage such conditions.

Osteocyte mechanobiology has been largely investigated using mouse cell lines like the MLO-Y4 instead of a human. This is due to the limited amount of bone material human volunteers can donate, tissue morbidity at the extraction site and technical difficulties around cell extraction and isolation from a hard matrix such as mineralised bone^19^. Although some molecular pathways involved in bone formation are conserved between mouse and human like the WNT/β-catenin^20^, not all are conserved. Indeed, it is well known that different species display different molecular networks linking genes to diseases^21^. This discrepancy led us to postulate that a portion of the signalling pathways and processes in osteocyte mechanotransduction may not be conserved across both species. Thus, another important aim of this work is to ascertain whether human and mouse osteocytic cells show differences from a transcriptome and secretome standpoint. By identifying potential differences, we will be able to make more informed decisions upon using human or mouse cells in basic and preclinical bone studies.

The transcriptome was mapped by RNA-seq which detected ~13,000 gene products in the murine cell line MLO-Y4 and ~14,000 in human stem cell-derived osteocytes (hSCO). The percentage of clean raw reads was homogenous, above 98% for every biological replicate (Supplementary Fig. 1). The hSCO showed 368 gene products exclusive to mechanically active and 619 to static (Fig. 1a). MLO-Y4 exhibited 286 gene products exclusive to mechanically active conditions and 242 exclusive to static (Fig. 1b). DESeq2 analysis revealed 821 differentially expressed gene transcripts in human and 13 in murine (Supplementary Fig. 2). Differentially expressed gene transcripts were analysed using the GO and the KEGG enrichment analysis. In stretched human cells, organelle, nucleus and intracellular were the most overrepresented biological processes while in stretched murine cells, transport, immune system process and extracellular region were the most enriched (Fig. 1c, d). Cell cycle, FoxO, pathways in cancer and DNA repair related pathways were the most significantly enriched signalling pathways in mechanically stretched hSCO whereas TNF, cytokine–cytokine interactions, PI3K-AKT, Jak-STAT, HIF-1 were mostly enriched in murine cells (Fig. 1e, f). Next, we profiled the protein secretome using tandem LC-MSMS. hSCO revealed 36 proteins exclusive to cyclic stretch and 95 proteins exclusive to static conditions (Fig. 2a) whereas murine cells revealed 124 proteins exclusive to mechanical loading and 166 exclusive to static conditions (Fig. 2b). The relations between proteins found exclusively under mechanically active and static conditions are depicted in Supplementary Figs. 3 (human) and 4 (mouse). The cellular location of all identified proteins was found similar under mechanically active and static conditions (Supplementary Fig. 5). Quantitative sequential windowed acquisition of all theoretical fragment ion mass spectra (SWATH-MS) analysis of mechanically stimulated cells revealed differential expression of 15 out of 379 secreted factors in hSCO (Fig. 2c) and 12 out of 841 in MLO-Y4 (Fig. 2d). GO enrichment analysis suggests that cholesterol or lipoprotein-related was the most significant biological process found in human cells (Fig. 2e) while in mouse it was response to oxidative stress (Fig. 2f). Process network enrichment analysis revealed that ECM remodelling, ossification and bone remodelling and cell–matrix interactions were the most significant processes found in stretched human cells. This was due to the differential expression of the proteins COOA1 (↑), SPARC (↓) and MFAP4 (↑) (Fig. 2g). Bone remodelling was also found to be significantly enriched in stretched murine osteocytic cells (Fig. 2h). Despite this similarity, in human cells, COAA1 and SPARC led to the enrichment of this process while in murine cells PRDX1 was the sole contributor. Human and murine heatmaps of differentially expressed proteins were found to aggregate into three distinct clusters (Supplementary Fig. 6).

**Fig. 1:**
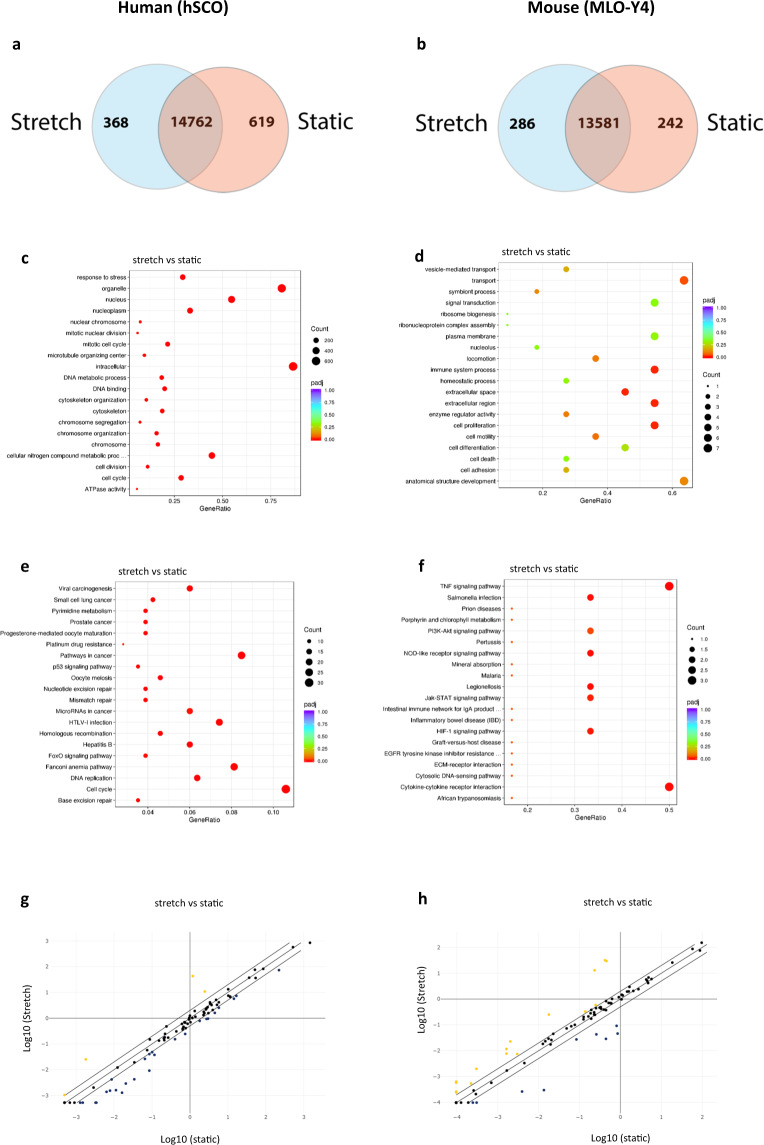
Transcriptome analysis and WNT signalling in murine and human osteocytic cells. Venn diagram of hSCO (**a**) and MLO-Y4 (**b**) showing the gene transcripts exclusive to mechanically active and static cell culture conditions; Chart depicting top 20 overrepresented biological processes in hSCO (**c**) and MLO-Y4 (**d**) with red dots indicating statistical significance; Chart of top 20 overrepresented signalling pathways in hSCO (**e**) and MLO-Y4 (**f**) with red dots indicating statistical significance; scatter plot of genes belonging to the WNT signalling pathways in hSCO (**g**) and MLO-Y4 (**h**); Gene expression was nonsignificant (*p* > 0.05); *n* = 3.

**Fig. 2:**
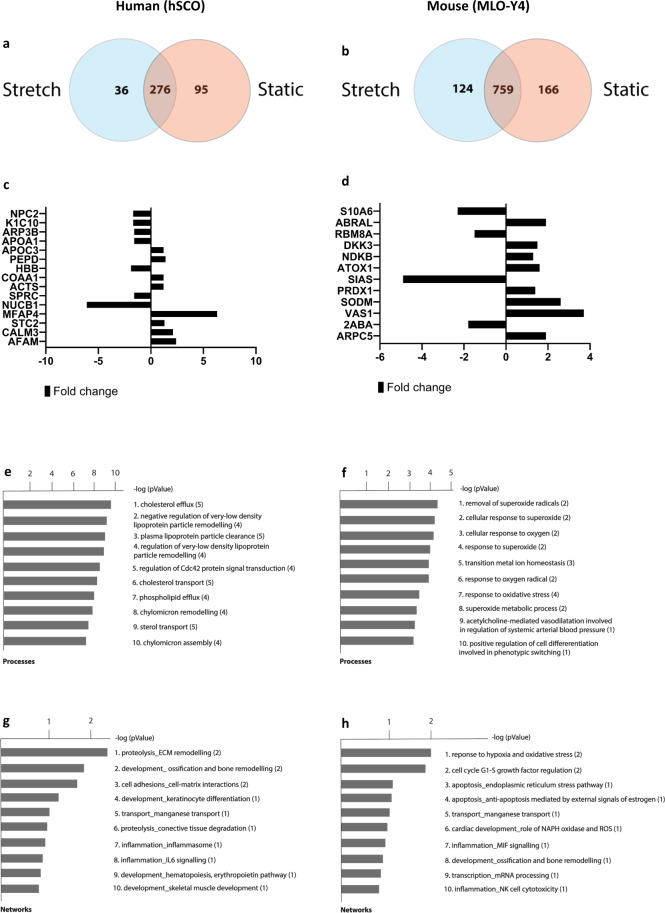
Secretome analysis of murine and human osteocytic cells. Venn diagram of hSCO (**a**) and MLO-Y4 (**b**) showing the secreted molecules exclusive to mechanically active and static cell culture conditions; Protein expression fold change given for the stretch condition vs control group (static) in hSCO (**c**) and MLO-Y4 (**d**); Chart depicting top ten overrepresented biological processes in hSCO (**e**) and MLO-Y4 (**f**); Chart depicting top ten overrepresented process networks in hSCO (**g**) and MLO-Y4 (**h**); Number of overexpressed proteins in each enrichment score within brackets; *n* = 4.

To gain further knowledge on signalling pathways governing bone tissue formation we searched for signs of WNT/β-activation in the transcriptome and secretome immediate post-loading. Analysis of the human transcriptome revealed a very modest but significant up-regulation of the WNT/β-catenin transcription factor, TCF7 (~0.7) (Supplementary Data 1). Analysis of 84 keys genes involved in the WNT signalling by RT-qPCR, suggests that gene expression remained unaltered immediate after loading in either human or murine osteocytic cells (Fig. 1g, h).

We successfully mapped the mechanosensitive gene transcripts and proteins secreted by human osteocytic cells after a single bout of cyclic stretch using RNA-seq and LC-MSMS, respectively. This allowed us to identify overrepresented signalling pathways, biological processes and network processes evoked by mechanically stimulated osteocyte-like cells likely to mediate bone regeneration or modulate cancer progression.

As mentioned earlier, mechanically stimulated osteocytes can influence bone regeneration through indirect bone remodelling which involves activation of PI3K/AKT and WNT signalling pathways, aiming to target downstream anabolic action provided by osteoblasts^7–9^. Our results suggest that in addition to this, osteocytes can modulate bone regeneration via direct remodelling of their extracellular environment since the network process ossification and bone remodelling was significantly enriched. This direct remodelling capacity is corroborated by a previous study on lactating mice demonstrating that osteocytes removed the ECM to potentially provide an extra source of calcium to the body^22^.

Exercise or mechanical stimulation of osteocytes appears to offer a protective effect against breast cancer progression^4,12,13^; however, the underpinning mechanisms remain unclear. We hypothesised that activation of signalling pathways linked to genome integrity could be one potential mechanism. This study confirmed our hypothesis since the signalling pathways cell cycle, P53, pathways in cancer and DNA-repair like mismatch repair and nucleotide excision repair, were all significantly enriched in stretched osteocytic cells. Another proposed mechanism by which mechanically stretched osteocytes may limit cancer progression is remodelling of the extracellular microenvironment, since ECM remodelling, ossification and bone remodelling and cell–matrix interactions were significantly enriched as mentioned earlier. Remodelling is likely to have an impact on this because cancer cells degrade the normal ECM to replace it with tumour ECM allowing the invasion of the cancer cells^23^. As mechanically stimulated osteocytic human cells remodel their extracellular environment this should compete against breakdown and deposition of tumour ECM and delay or avoid cancer invasion. Although host–tumour interactions are gaining increasing attention in exercise and cancer^24^, functional studies involving cocultures of mechanically stimulated osteocytic cells with cancer cells would be needed to determine their contribution to cancer progression limitation.

A comparative analysis across human and mouse osteocytic cell lines evidenced that the transcriptome and secretome were quite distinctive between the two cell lines. KEGG analysis of the human transcriptome identified as potential mechanosensitive signalling pathways, cell cycle, pathways in cancer, P53, DNA repair related pathways and FoxO, whereas examination of the mouse identified TNF, cytokine–cytokine receptor interactions and PI3K-AKT. As for secretome, two network processes were significantly enriched out of the top ten in both cell lines—ossification and bone remodelling and manganese transport; however, the proteins leading to such enrichment were different. We speculate that variances found between both osteocytic cell lines are because of fundamental differences between the two—one is human and derives from a stem cell model and the other is a mouse immortalised transgenic cell line.

Genetic activation of osteocyte WNT/β-catenin is vital for bone healing^25^. In mouse, WNT/β-catenin activation typically occurs within 1–24 h post-loading (at rest) as demonstrated by β-galactosidase positive staining and increased gene expression of Wnts 1 and 7^14,15^. In the osteocytic MLO-Y4 cell line, gene expression of WNT-related gene Wnt3a is upregulated at 1–3-h post-stimulation with pulsatile FFSS (at rest)^26^. To ascertain whether 1 bout of cyclic stretch was enough to activate WNT/β-catenin signalling in human or mouse osteocytic cells, the relative expression of 84 key genes of the WNT/β-catenin signalling was analysed by RT-qPCR immediate post-loading. RT-qPCR showed that WNT/β-catenin gene expression remained unchanged. A closer analysis of RNA-seq and LC-MSMS indicated potential signs of activation, since the β-catenin target, TCF7 was slightly upregulated in human osteocytic cells and the signalling pathways PI3K-AKT which crosstalk with WNT/β-catenin was enriched in the mouse cells. However, we were expecting to detect a more robust response in preparation of the above events related to WNT/β-catenin activation, in particular, the significant enrichment of the WNT/ β-catenin signalling pathway. Given that we analysed WNT/β-catenin immediate after cyclic stretch and did not found a robust response and other studies examining 1–3 up to 24 h post-loading (at rest) did, we postulate that a rest period after mechanical stimulation is fundamental to trigger strong activation of WNT/β-catenin signalling in osteocytes.

To summarise, this study suggests that mechanically stimulated osteocytes support bone regeneration via ossification and ECM remodelling and modulate cancer through transcriptional activity linked to genome integrity and ECM remodelling. Transcriptomic and secretomic data show dissimilar results between human and mouse cell lines in response to the same cyclic stretch regimen suggesting that mouse cells could be suboptimal predictors of human response to non-pharmacological interventions like exercise and should only be used when the signalling pathways or processes to be investigated are highly evolutionary conserved.

Despite the importance of these findings, we do recognise the importance of repeated bouts of mechanical loading and the performance of functional studies involving stretched osteocytes and cancer cells. Therefore, future studies addressing both aspects are warranted. As our understanding of the transcriptome and secretome in stretched cells and human bone cell models progresses, RR protocols based on exercise may be efficiently translated to the clinic.

## Methods

### Cell culture

Human osteocyte-like cells derived from a stem cell culture model: Adipose stem cells (ASCs) were purchased from ATCC (ATCC^®^ PCS -500-011^™^) and expanded in complete growth medium (ATCC^®^ PCS-500-030^™^ and ATCC^®^ PCS-500-040^™^) supplemented with 1% penicillin-streptomycin until reaching 70–80% confluence and passaged to a 6-well plate at a cell density of 18,000 viable cells/cm^2^. After 48 h, the complete growth medium was removed, cells were washed twice with PBS and differentiation medium (ATCC^®^ PCS-500-052^™^) was added. The differentiation medium was changed every 3–4 days. ASCs differentiated between 13 and 15 days. Osteocyte-like cells derived from ASCs stained positive for Alizarin red, FGF23 and exhibited dendritic-like extensions like primary osteocytes.

MLO-Y4: This cell line was acquired from Kerafast (EKC002) and expanded in MEM-α (Fisher) supplemented with 2.5% foetal calf serum (FBS) and 2.5% newborn calf serum (NCS) both inactivated (Fisher) and 1% penicillin-streptomycin until reaching 70% confluence. The medium was changed every 2–3 days. Both cell lines were cultured in a humified incubator at 37 °C and 5% CO_2_.

### Mechanical loading

Multiple studies reported that a single bout of exercise is enough to observe positive regenerative and health benefits, e.g. skeletal muscle satellite cells numbers increase^27^, and vascular function is normalised^28^ and therefore we opted for a single bout of mechanical loading rather than a multi bout one.

Upon exercise, bone experiences a myriad of forces, tension, compression, torsion and FFSS. A study comparing FFSS with cyclic tension suggests that human-derived bone cells react differently to both stimuli and that tension is a more potent stimulus for bone formation than FFSS as evidenced by the increased production of the type I collagen^29^. Due to the anabolic action of tension and its importance in RRPs, we decided to culture osteocytic cells under cyclic stretch rather than FFSS.

Briefly, mouse and human osteocytic cells were plated in a collagen-coated flexible bottom 6-well plate (Biopress, Flexcell) with a density of 15,000 viable cells/cm^2^ for 24 h. The plates were transferred to the bioreactor (Flexcell) to initiate the mechanical loading protocol, 34,000 μS, 2 Hz for 5 h^30,31^. Cells seeded on the same plates but cultured under static conditions were used as control. Mechanically loaded and static osteocyte-like cells were cultured in a humidified incubator at 37 °C and 5% CO_2_.

### RNA extraction

Immediately after the mechanical loading, cells were washed twice with ice-cold PBS and lysed with RLT buffer (Qiagen) for 1 min. Next, RNA was extracted using the RNeasy kit (Qiagen) according to the manufacturer instructions. RNA quantity and quality were estimated using the NanoDrop™ 2000 spectrophotometer (Thermo Scientific™).

### cDNA synthesis and quantitative gene expression

Gene expression was analysed by RT-qPCR. After RNA extraction, cDNA was synthesised using the RT² First Strand Kit (Qiagen) according to the manufacturer instructions. As for the RT-qPCR, the RT2 Profiler^™^ PCR Array Human WNT Signalling Pathway PAHS-043Y (Qiagen) and the RT² Profiler^™^ PCR Array Mouse WNT Signalling Pathway PAMM-043Z (Qiagen) were used to quantify relative expression genes of the WNT signalling pathway. RT-qPCR was performed in the Rotorgene 6000 thermocycler (Corbett Research) using the SYBR Green ROX master mix (Qiagen) following the manufacturer instructions. CT values were exported to an Excel file to create a table of CT values and this table uploaded to the data analysis web portal at http://www.qiagen.com/geneglobe. CT values were normalised based on an automatic selection from a full panel of reference genes. Fold-Change (2^^(−Delta Delta CT)^) was calculated based on the normalised gene expression (2^^(−Delta CT)^) in the mechanically stimulated samples divided by the normalised gene expression (2^^(−Delta CT)^) in the control sample. The Student’s *t*-test (two-tailed) was used to perform comparisons between stretched cells and static control. Three independent experiments were performed.

### RNA-seq

RNA degradation and contamination were monitored on 1% agarose gels. RNA purity was checked using the NanoPhotometer^®^ spectrophotometer (IMPLEN, CA, USA). RNA integrity and quantitation were assessed using the RNA Nano 6000 Assay Kit of the Bioanalyzer 2100 system (Agilent Technologies, CA, USA). A total amount of 1 µg RNA per sample was used as input material for the RNA sample preparations. Sequencing libraries were generated using NEBNext^®^ UltraTM RNA Library Prep Kit for Illumina^®^ (NEB, USA) following the manufacturer’s recommendations and index codes were added to attribute sequences to each sample. Briefly, mRNA was purified from total RNA using poly-T oligo-attached magnetic beads. Fragmentation was carried out using divalent cations under elevated temperature in NEBNext First Strand Synthesis Reaction Buffer (5X). First-strand cDNA was synthesised using random hexamer primer and M-MuLV Reverse Transcriptase (RNase H-). Second strand cDNA synthesis was subsequently performed using DNA Polymerase I and RNase H. Remaining overhangs were converted into blunt ends via exonuclease/polymerase activities. After adenylation of 3′ ends of DNA fragments, NEBNext Adaptor with hairpin loop structure was ligated to prepare for hybridisation. To select cDNA fragments of preferentially 150–200 bp in length, the library fragments were purified with AMPure XP system (Beckman Coulter, Beverly, USA). Then 3 µl USER Enzyme (NEB, USA) was used with size-selected, adaptor-ligated cDNA at 37 °C for 15 min followed by 5 min at 95 °C before PCR. Then PCR was performed with Phusion High-Fidelity DNA polymerase, Universal PCR primers and Index (X) Primer. At last, PCR products were purified (AMPure XP system) and library quality was assessed on the Agilent Bioanalyzer 2100 system. The clustering of the index-coded samples was performed on a cBot Cluster Generation System using PE Cluster Kit cBot-HS (Illumina) according to the manufacturer’s instructions. After cluster generation, the library preparations were sequenced on an Illumina NovaSeq 6000 S4 platform and paired-end reads were generated. Reference genome and gene model annotation files were downloaded from the genome website browser (NCBI/UCSC/Ensembl) directly. Paired-end clean reads were mapped to the reference genome using HISAT2 software. Differential expression analysis between stretch and static control was performed using the DESeq2 R package. The resulting *P* values were adjusted using the Benjamini and Hochberg’s approach for controlling the false discovery rate (FDR). Genes with an adjusted *P* value < 0.05 found by DESeq2 were assigned as differentially expressed. Three independent experiments were performed.

### Secretome and proteomic quantitative mass spectrometry

Osteocyte-like cells were cultured in a collagen-coated flexible bottom 6-well plate (Bioflex, Flexcell) with a cell density of 18,000 viable cells/cm^2^ in serum-free conditions (MEM-α, Fisher), overnight. Culture medium (secretome) was collected immediately after the mechanical loading, centrifuged at 300 × *g* for 5 min to pellet cells and debris, filtered through a 0.22 µm filter and concentrated using a 5 kDa cut-off centrifugal concentrator unit (Sartorius). One hundred micrograms of protein were incubated with 8.5 M urea, 2% DDT and 1% *N*-octyl-beta-glucopyranoside for 30 min at room temperature. A total of 50 μg of protein were processed and digested (trypsin) using S-trap micro methodology (Protifi). Samples were dried and kept at −80 °C until analysis. On the day of analysis, samples were resuspended in 5% acetonitrile and 0.1% formic acid. Peptides/proteins were analysed by quantitative mass spectrometry (TripleTOF 6600, SCIEX) using SWATH and IDA and processed using OneOmics (SCIEX) software as described previously^32,33^. The method used for normalisation of the data was the most likely ratio normalisation^33^. 51% confidence limit and a minimum of two peptides per protein were used as criteria for assessing fold change. Four independent experiments were performed.

### Bioinformatics

#### Transcriptome

GO enrichment analysis of differentially expressed genes was implemented by the GOseq R package, in which gene length bias was corrected. GO terms with corrected *P* value less than 0.05 were considered significantly enriched by differential expressed genes. We used KOBAS software to test the statistical enrichment of differential expression genes in KEGG pathways.

#### Secretome

Biological processes of significantly changed proteins were analysed by gene ontology using the curated database Metacore (Clarivate Analytics). The percentage of overlap between proteins secreted from mechanically stimulated and static cells was estimated using the Venny 2.1 software. Protein interaction maps were generated using the Cytoscape STRING App and String DB. Protein location was estimated using Panther^™^.

### Reporting summary

Further information on research design is available in the Nature Research Reporting Summary linked to this article.

## Supplementary information

Supplementary Data 1

Supplementary Information

Reporting Summary

## Data Availability

The mass spectrometry proteomics data have been deposited to the ProteomeXchange Consortium (http://proteomecentral.proteomexchange.org) via the PRIDE partner repository^34^ with the dataset identifier PXD020774. Transcriptomic data are available in the ArrayExpress database^35^ (http://www.ebi.ac.uk/arrayexpress) under accession number E-MTAB-9487.
