# Supplementary material for: Multiomic analysis of stretched osteocytes reveals processes and signalling linked to bone regeneration and cancer
Source: NPJ Regen Med. 2021 Jun 7;6:32. doi: 10.1038/s41536-021-00141-3 (PMC8184808; doi:10.1038/s41536-021-00141-3)
Supplement: Supplementary file 2 — Supplementary Information [file 41536_2021_141_MOESM2_ESM.pdf]

Human

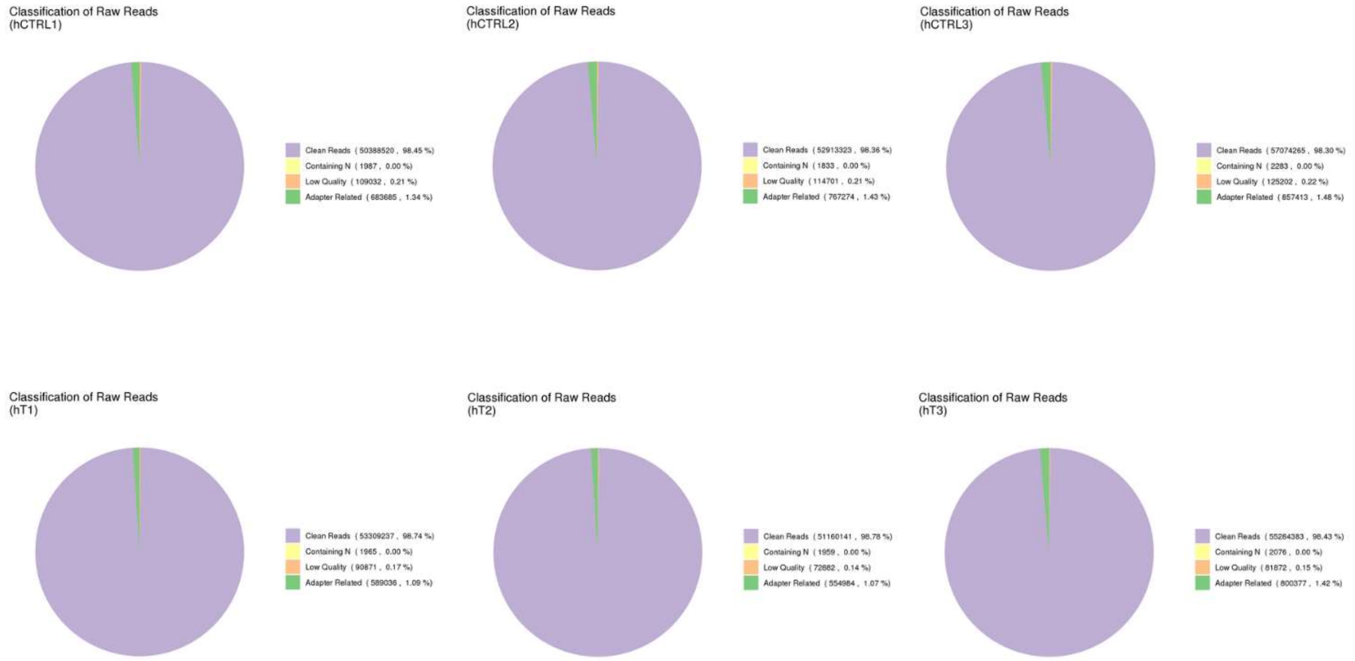

Mouse

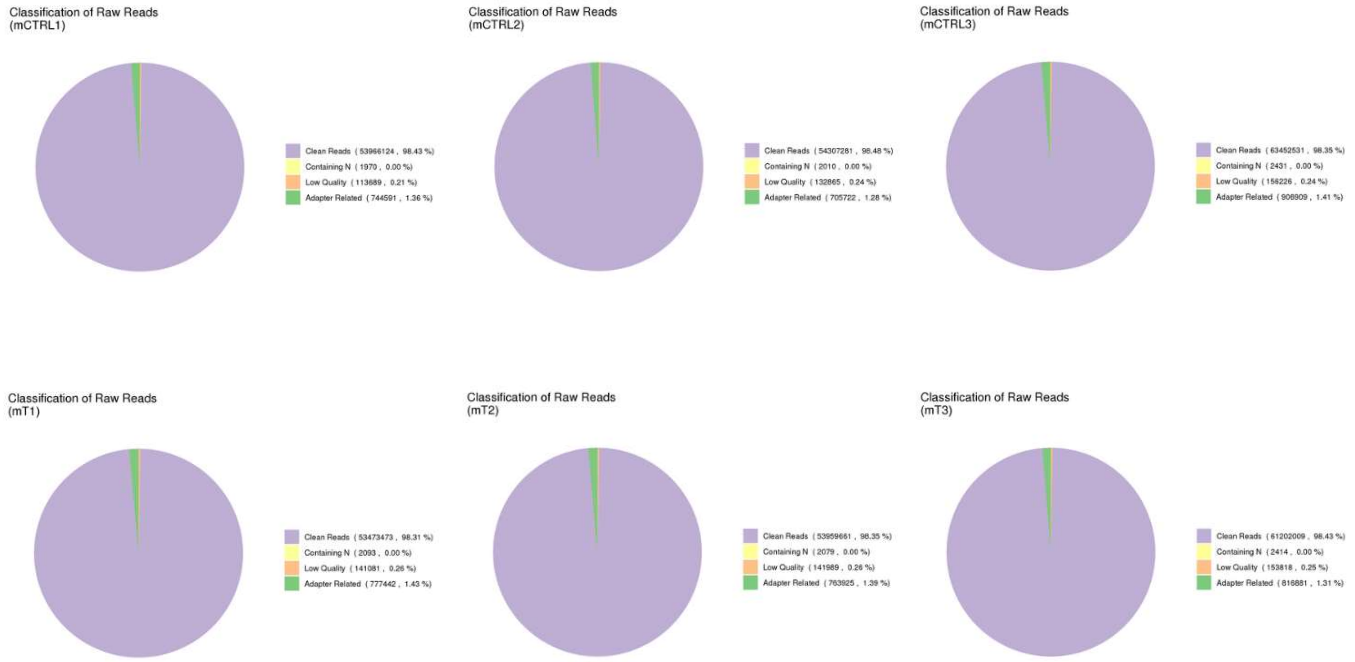

Supplementary Figure 1. Percentage of clean raw reads obtained from each human or mouse cell sample showing a homogeneous distribution; n=3

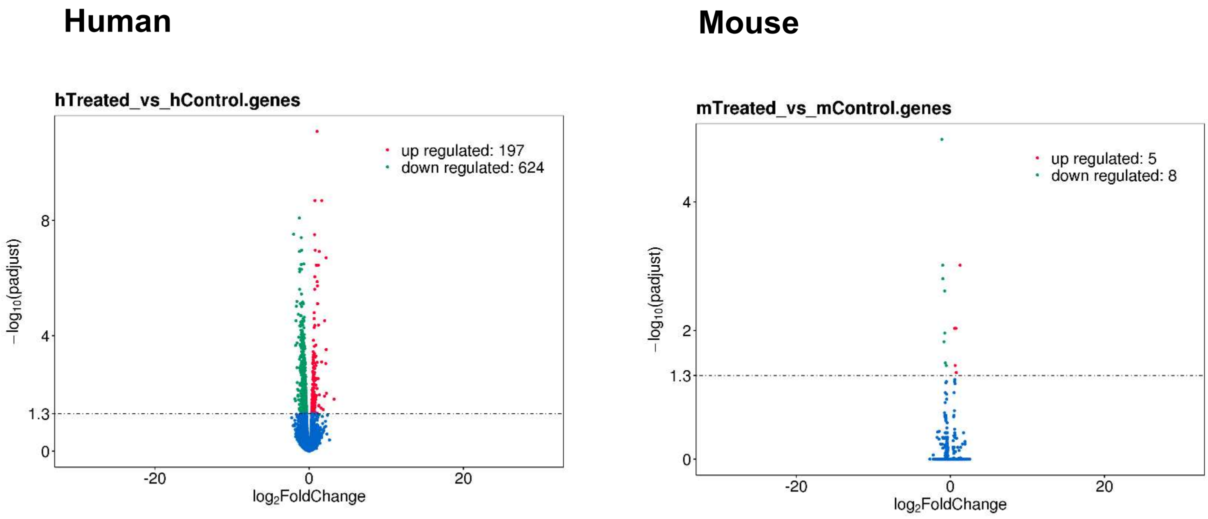

Supplementary Figure 2. Number of differentially expressed gene transcripts in human and mouse cell lines; n=3

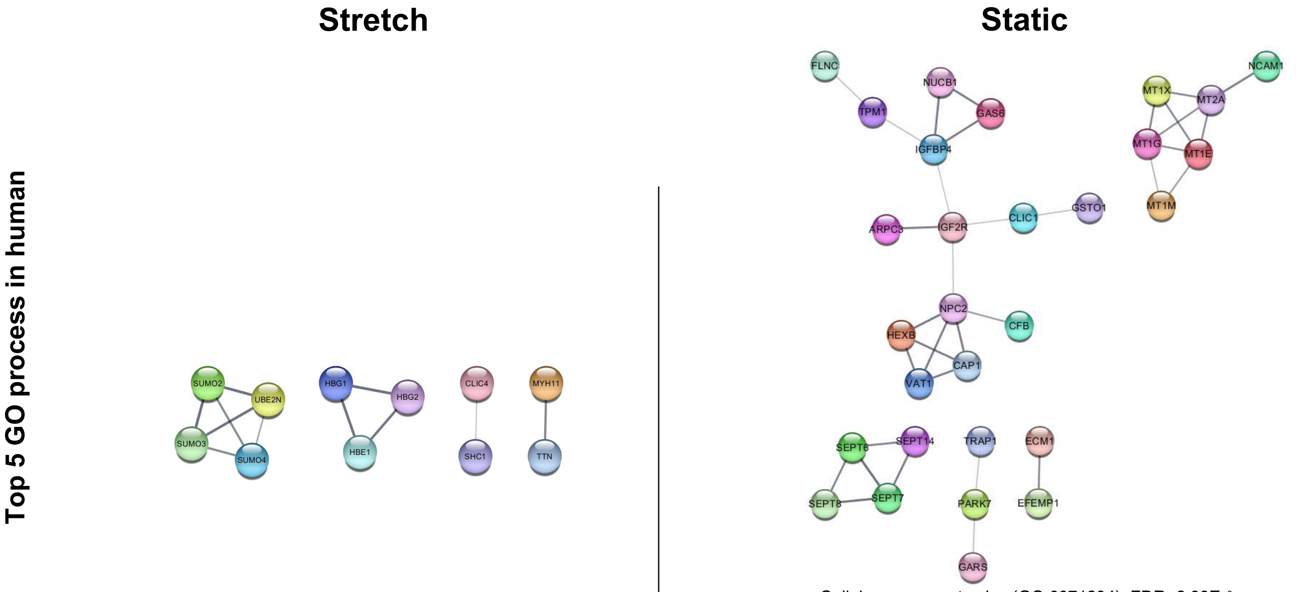

Oxygen transport (GO: 0015671) FDR=9.50E $^{-4}$
Protein sumoylation (GO: 0016925) FDR=0.0032
Skeletal muscle myosin thick filament (GO: 0030241) FDR=0.0032
Actin cytoskeleton organisation (GO: 0030036) FDR=0.0107
Activation of MAPK activity (GO: 0000187) FDR=0.0264

Cellular response to zinc (GO:0071294) FDR=2.99E $^{-6}$
Response to stimulus (GO: 0050896) FDR=6.50E $^{-4}$
Import into cell (GO: 0098657) FDR=0.0028
Immune response (GO: 006955) FDR= 0.052
Negative regulation of intrinsic apoptotic signaling pathway in response to hydro - gen peroxide (GO: 1903751) FDR=0.0106

Supplementary Figure 3. Protein relations identified in the secretome of human osteocytes derived from a stem cell culture model cultured under stretch and static conditions and respective top 5 GO processes. The secretome of stretched cells exhibited a cluster of 3 SUMO proteins (implicated in post-translation modifications), whereas the one from cells cultured under static conditions depicted a cluster of 4 SEPT proteins (implicated in cell division); Proteins with no relations found were excluded; n=4

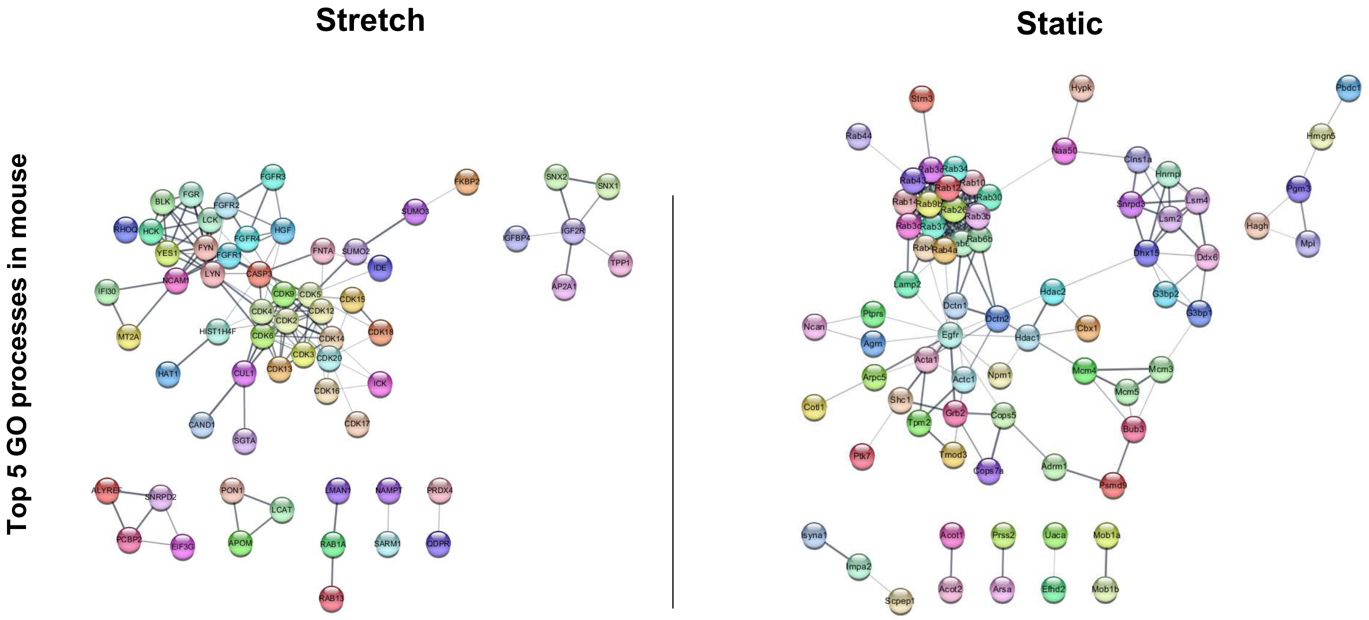

Protein phosphorylation (GO: 0006468) FDR=1.56E $^{-16}$
Nitrogen compound metabolic process (GO: 0006807) FDR=3.95E $^{-11}$
Transmembrane receptor protein tyrosine kinase signaling pathway (GO: 0007169) FDR=3.95E $^{-11}$
Regulation of cell population proliferation (GO: 0042127) FDR=9.18E $^{-09}$
Peptidyl-amino acid modification (GO: 0018193) FDR=2.13E $^{-08}$

Cellular component organization or biogenesis (GO: 0071840) FDR=4.74E $^{-05}$
Vesicle-mediated transport (GO: 0016192) FDR=2.90E $^{-04}$
Antigen processing and presentation (GO: 0019882) FDR=2.90E $^{-04}$
Peptidyl-cysteine methylation (GO: 0018125) FDR=6.20E $^{-04}$
Regulation of vesicle-mediated transport (GO: 0060627) FDR=6.20E $^{-04}$

Supplementary Figure 4. Protein relations identified in the secretome of the mouse cell line MLO-Y4 cultured under stretch and static conditions and respective top 5 GO processes. The secretome from stretched cells exhibited a large cluster of 14 CDKs proteins (implicated in the cell cycle), whereas the one from cells cultured under static conditions depicted a large cluster of 17 Rab proteins (involved in protein transport); Proteins with no relations found were excluded; n=4

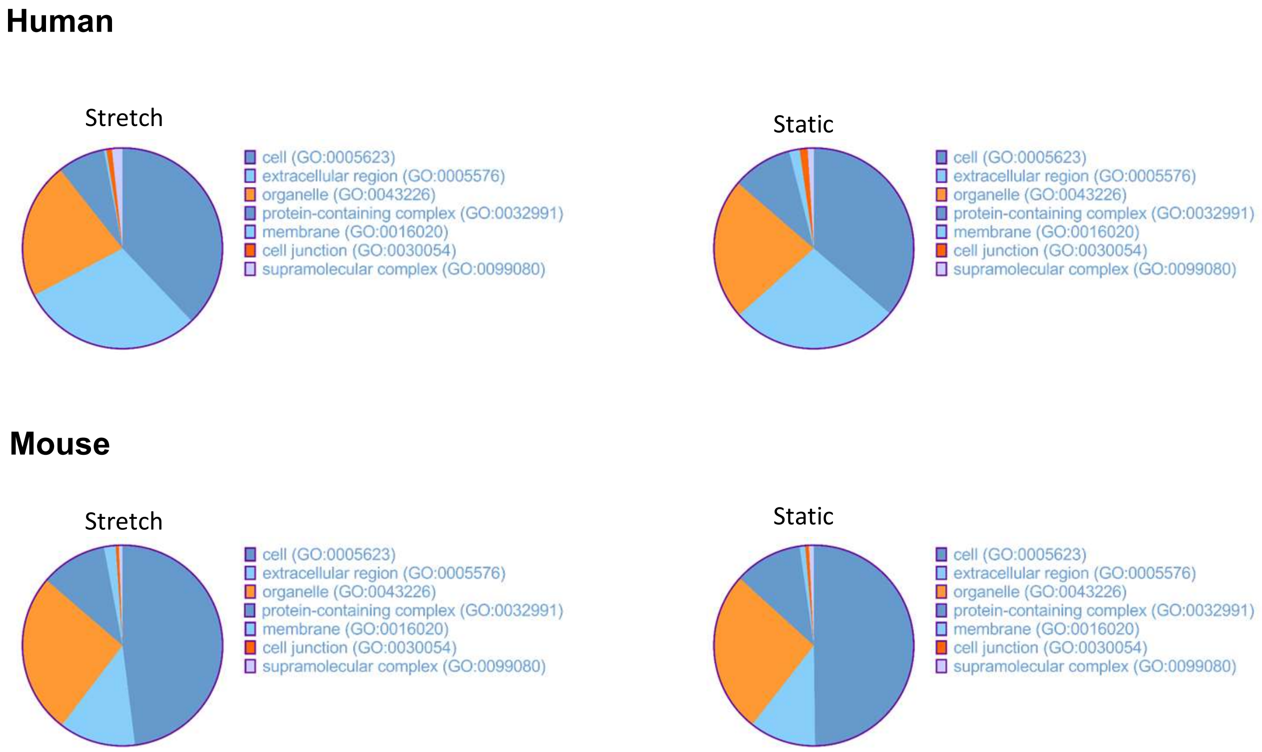

Supplementary Figure 5. Cellular location of proteins identified in human and mouse osteocytic cells under stretch or static conditions; n=4

Human **Mouse**

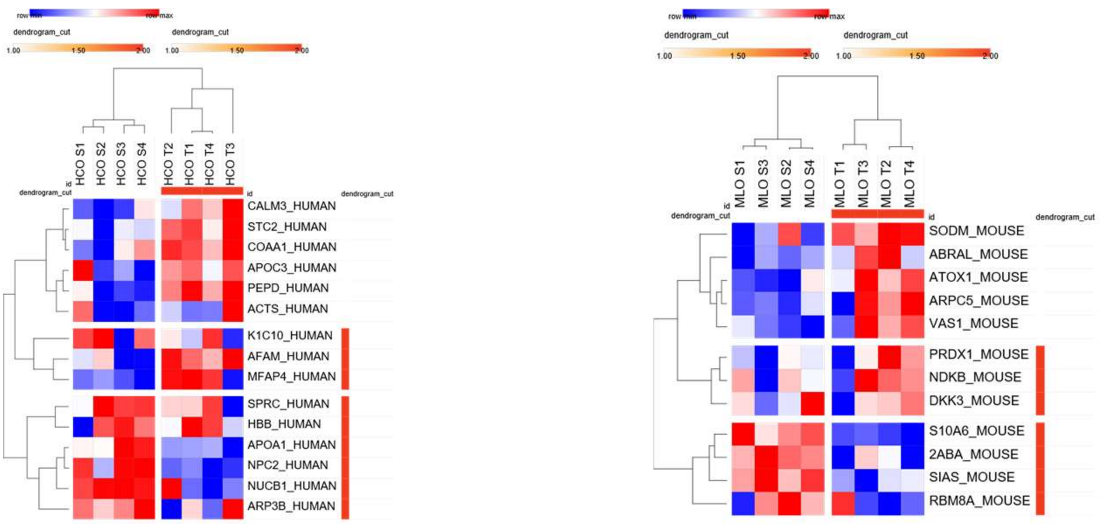

Supplementary Figure 6. Human and mouse heatmaps displaying non-supervised clusters of differentially expressed proteins; n=4

Supplementary information on RNA-seq, LC-MSMS and bioinformatic analysis
